# Monitoring HSVtk suicide gene therapy: the role of [^18^F]FHPG membrane transport

**DOI:** 10.1038/sj.bjc.6602216

**Published:** 2004-12-21

**Authors:** A R Buursma, I J van Dillen, A van Waarde, W Vaalburg, G A P Hospers, N H Mulder, E F J de Vries

**Affiliations:** 1 PET Center, Groningen University Hospital, PO Box 30.001, Hanzeplein 1, 9700 RB Groningen, The Netherlands; 2 Department of Medical Oncology, Groningen University Hospital, PO Box 30.001, 9700 RB Groningen, The Netherlands; 3 Department of Pathology, Groningen University Hospital, PO Box 30.001, 9700 RB Groningen, The Netherlands

**Keywords:** positron emission tomography, gene therapy, herpes simplex virus thymidine kinase, ganciclovir, [^18^F]FHPG, membrane transport, acyclovir

## Abstract

Favourable pharmacokinetics of the prodrug are essential for successful HSVtk/ganciclovir (GCV) suicide gene therapy. [^18^F]FHPG PET might be a suitable technique to assess the pharmacokinetics of the prodrug GCV noninvasively, provided that [^18^F]FHPG mimics the behaviour of GCV. Since membrane transport is an important aspect of the pharmacokinetics of the prodrug, we investigated the cellular uptake mechanism of [^18^F]FHPG in an HSVtk expressing C6 rat glioma cell line and in tumour-bearing rats. The nucleoside transport inhibitors dipyridamol, NBMPR and 2-chloroadenosine did not significantly affect the [^18^F]FHPG uptake *in vitro*. Thymidine and uridine significantly decreased [^18^F]FHPG uptake by 84 and 58%, respectively, but an enzyme assay revealed that this decline was due to inhibition of the HSVtk enzyme rather than membrane transport. Nucleobase transport inhibitors, thymine and adenine, caused a 58 and 55% decline in tracer uptake, respectively. *In vivo*, the ratio of [^18^F]FHPG uptake in C6tk and C6 tumours decreased from 3.0±0.5 to 1.0±0.2 after infusion of adenine. Thus, in our tumour model, [^18^F]FHPG transport exclusively occurred via purine nucleobase transport. In this respect, FHPG does not resemble GCV, which is predominantly taken up via the nucleoside transporter, but rather *acyclovir*, which is also taken up via the purine nucleobase carrier.

Suicide gene therapy offers a potential treatment modality for various forms of cancer. Hitherto, the herpes simplex virus type 1 thymidine kinase gene (HSVtk) is the most frequently studied suicide gene. Expression of the HSVtk gene renders tumour cells sensitive to antiviral agents like ganciclovir (GCV). Compared to human thymidine kinase, HSVtk is less substrate specific and therefore can efficiently convert GCV to its monophosphate, which is phosphorylated further to the corresponding di- and triphosphate by host cellular kinases (Cheng *et al*, 1983). These metabolites are toxic for the cells and consequently tumour cells in which the HSVtk gene is expressed are killed (Culver *et al*, 1992; Freeman *et al*, 1993). However, clinical results remain disappointing because the conditions for clinical gene therapy protocols are not yet optimal. Low efficiency of gene transfer to the target cells seems to be the major problem. Another important issue is the safety profile of the therapy: in general, invasion of the gene transduction vector in nontarget organs can induce serious toxicity (Somia and Verma, 2000). To optimise HSVtk gene therapy, an *in vivo* detection method for HSVtk activity would be extremely helpful. Positron Emission Tomography (PET) imaging with a suitable tracer can provide useful information about the location and the extent of the HSVtk enzyme activity as a function of time.

Several radiolabelled thymidine and GCV derivatives have been studied as potential tracers for imaging of HSVtk enzyme activity with PET. After phosphorylation, these compounds remain trapped within cells and are therefore suitable for imaging. We and others have shown that HSVtk gene expression can successfully be monitored with 9-[(3-[^18^F]fluoro-1-hydroxy-2-propoxy)methyl]guanine ([^18^F]FHPG) and PET (Alauddin *et al*, 1999; Hospers *et al*, 2000). Recently, other radiolabelled nucleoside analogues – especially 2′-fluoro-2′-deoxy-1-*β*-D-arabinofuranosyl-5-iodouracil (FIAU) – have shown to be more sensitive probes for HSVtk expression than [^18^F]FHPG, as FIAU accumulation in HSVtk transduced tumours was up to 119 times higher than that of FHPG (Brust *et al*, 2001; Tjuvajev *et al*, 2002).

The observed difference in sensitivity between FIAU and FHPG cannot be explained by differences in affinity for the HSVtk enzyme alone. The affinities of several nucleoside analogues for HSVtk have previously been determined in thymidine kinase assays using purified HSVtk enzyme. Cheng *et al* (1981) showed that the relative phosphorylation rates of the thymidine derivatives 2′-fluoro-2′-deoxy-1-*β*-D-arabinofuranosyl-5-methyluracil (FMAU) and FIAU (expressed as percentage of the phosphorylation rate of thymidine) were 42 and 104%, respectively. In another HSV-1 strain, Martin *et al* (1986) showed that phosphorylation rates of the acyclic guanosine analogues GCV and FHPG were 98 and 67% relative to thymidine, respectively. These results indicate that the affinities of GCV, FHPG, FMAU and FIAU for the HSVtk enzyme are comparable and cannot explain the large difference between FIAU and FHPG accumulation in HSVtk transduced tumours. Apparently, other factors than the affinity for the HSVtk enzyme play an important role in tracer/prodrug uptake.

This hypothesis is in agreement with the results of Beck *et al* (1995), who found that different tumour cells that expressed similar levels of HSVtk could display highly different susceptibilities to GCV *in vitro*. Similar results were obtained *in vivo*: mammary adenocarcinomas (TSA-tk tumours) could be eliminated in almost all animals by systemic GCV administration (150 mg kg^−1^ GCV i.p. twice daily during 5 days), whereas T-cell lymphomas (ESB-tk tumours) were completely resistant to GCV treatment. Haberkorn *et al* (1998) suggested that membrane transport might be a limiting factor for HSVtk/GCV suicide gene therapy, as tritiated GCV enters the cell mainly via relatively slow active membrane transport. Therefore, for successful HSVtk/GCV suicide gene therapy, not only the level of suicide gene expression but apparently also the rates of uptake and conversion of the prodrug into the toxic substance, the prodrug pharmacokinetics, are important. To discriminate between responders and nonresponders in an early stage of treatment, a method to noninvasively measure the pharmacokinetics of the prodrug would be helpful. The dynamics of various enzyme systems have been studied with PET and a suitable radiolabelled compound. The best-known example is probably [^18^F]fluorodeoxyglucose, that is used to study the glucose metabolism. In a similar manner, [^18^F]FHPG PET could be of benefit to study HSVtk prodrug pharmacokinetics.

In clinical studies on HSVtk suicide gene therapy, GCV has been used as the prodrug. Thus far, a suitable method to label GCV with a PET isotope (i.e. ^11^C) is not available. Owing to the structural resemblance between FHPG and GCV (Figure 1

**Figure 1:**
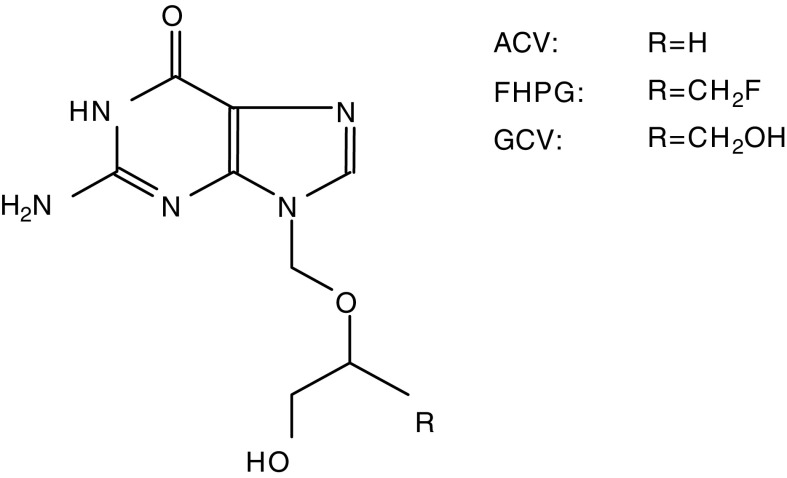
The chemical structures of GCV, FHPG and ACV.

), we hypothesised that [^18^F]FHPG PET could be applied to assess the pharmacokinetics of the prodrug (de Vries *et al*, 2003). However, the replacement of the -OH group in the structure of GCV with -F does not guarantee identical pharmacokinetic properties. Therefore, the pharmacokinetic profile of FHPG remains to be investigated.

It has already been shown that the phosphorylation characteristics of HSVtk for the substrates GCV and FHPG are comparable (Martin *et al*, 1986). However, pharmacokinetics are not only determined by phosphorylation but also by transport of the drug from plasma into the cell. At present, little is known about transport of FHPG across the cell membrane. To investigate whether the transport mechanism of FHPG resembles that of GCV, this study aimed to elucidate the transport mechanism of FHPG.

## MATERIALS AND METHODS

### Materials

Dulbecco's minimum essential medium (DMEM), geneticin (G418), trypsin (2.5% (w v^−1^)) and bovine serum albumin (BSA) were purchased from Invitrogen (Merelbeke, Belgium). Foetal calf serum (FCS) and Matrigel were obtained from PAA laboratories (Brunschwig, Amsterdam, The Netherlands). Thymidine, uridine, 2-chloroadenosine, thymine, adenine, dipyridamole, nitrobenzylthioinosine (NBMPR), dithiothreitol (DTT) and aprotinin were obtained from Sigma-Aldrich (St Louis, MO, USA). GCV was obtained from Roche (Mijdrecht, The Netherlands). Tris, potassium chloride (KCl), potassium dihydrophosphate, dipotassium hydrophosphate, magnesium chloride (MgCl_2_), glycerol, isopropanolol, phenylmethylsulphonyl fluoride (PMSF) and Titriplex (EDTA) were purchased from Merck (Darmstadt, Germany). Phosphate-buffered saline (PBS) and sodium fluoride (NaF) were purchased from the Department of Pharmacy (Groningen University Hospital, Groningen, The Netherlands). Adenosine triphosphate (ATP) was obtained from Pharmacia BV (Woerden, The Netherlands). Whatman ion-exchange DE-81 filter paper disks were purchased from VWR International (Roden, The Netherlands).

### Preparation of [^18^F]FHPG

[^18^F]FHPG was prepared as described by Alauddin *et al* (1996) with minor modifications. The fluorination reaction was carried out at 125°C for 30 min and the subsequent hydrolysis was performed at 90°C for 5 min. The reaction mixture was neutralised by the addition of sodium phosphate buffer (pH 7.2) prior to purification by high-performance liquid chromatography (HPLC), which was performed over a semipreparative Alltima C18 reverse-phase column (5 *μ*m, 10 × 250 mm^2^. Alltech, Deerfield, IL, USA) using 3% EtOH, 10 mM NaH_2_PO_4_ in saline as the eluent (flow: 5 ml min^−1^, *R*_*t*_: 16 min). Radiochemical yield: 5–15% (corrected for decay). Radiochemical purity >99% (HPLC: Nova Pak C18, 3 *μ*m, 9 × 150 mm, 2% acetonitrile, 1 ml min^−1^, *R*_*t*_: 6 min).

### Cell lines

C6 rat glioma cells obtained from the American Type Culture Collection were cultured in monolayers in DMEM supplemented with 5% FCS in a humidified atmosphere with 5% CO_2_ at 37°C. HSVtk positive C6 cells (C6tk) were obtained by transfection of the C6 cells with supernatant of PA-317tk packaging cells containing replication incompetent retroviruses carrying the HSVtk gene and the NeoR gene. Subsequent G418 selection resulted in the C6tk cell line. Stable resistance in the C6tk cells was assured by culturing this cell line in the presence of G418 (0.5 mg ml^−1^). The packaging cell PA-317tk was a gift from Dr SG Marcus (Genetic Therapy Inc., Gaithersburg, MD, USA).

### Preparation of crude cell lysates

Approximately 1.5 × 10^7^ cells were trypsinised and washed twice with cold PBS, after which the cells were kept on ice and lysed in 0.5 ml lysis buffer (20 mM Tris buffer, pH 8.1, 40 mM KCl, 1 mM EDTA, 1 mM DTT, 1 mM PMSF, 1 *μ*g ml^−1^ aprotinin and 10% glycerol) (Hinds *et al*, 2000) by sonification (Soniprep 150, MSE, Leicestershire, UK) for 4–6 times 15 s. The homogenate was centrifuged at 15 000 **g** for 20 min to separate insoluble material. Protein concentrations were determined according to Bradford (1976) and lysates were diluted to a concentration of 5 mg protein ml^−1^ and stored at −80°C.

### Accumulation of [^18^F]FHPG in tumour cells

C6 cells and C6tk cells were seeded in 12-well culture plates with 1.5 ml of DMEM supplemented with 5% FCS. After 24 h at 37°C, monolayers had grown. The medium was removed and replaced with 1.5 ml of DMEM supplemented with either the inhibitors of the equilibrative facilitated diffusion nucleoside transport systems dipyridamole and NBMPR (both to a final concentration of 5 *μ*M), the substrates of the concentrative sodium-dependent nucleoside transporters 2-chloroadenosine, thymidine and uridine, the nucleobase transport inhibitors thymine and adenine, or with GCV (all to a final concentration of 2 mM). After 20 min of preincubation with these transport inhibitors at 37°C, 2.4±1.1 MBq of [^18^F]FHPG was added to each well. After 20 min of tracer incubation, the culture medium was removed and the monolayers were washed three times with 1 ml PBS. The cells were harvested from the culture plates by treatment with 0.25 ml trypsin for approximately 5 min. When the cells were detached from the bottom of the well, the cells were resuspended in 1.2 ml of culture medium to neutralise the trypsin. A 50 *μ*l sample was taken and mixed with 50 *μ*l trypan blue to count the number of viable cells under a microscope. The radioactivity in the cell suspensions was measured in a gamma counter (LKB, Wallac, Turku, Finland) and normalised to the number of viable cells. Results are reported as the percentage [^18^F]FHPG uptake relative to the [^18^F]FHPG uptake in C6tk cells that were not pretreated with a transport inhibitor. For each transport inhibitor, at least three independent experiments were performed. For GCV, two independent experiments were performed in duplicate and triplicate, respectively. Statistical analysis was performed on the raw data (*n*=5–12), using the two-sided unpaired student's *t*-test. *P*<0.05 was considered significant.

### Enzyme assay

The activity of the HSVtk enzyme in the crude cell lysates was determined as described by Hinds *et al* (2000), using [^18^F]FHPG as a substrate. The following standard reaction mixture was used: 100 *μ*l of crude cell lysate, 20 mM potassium phosphate (pH 7.6), 40 mM KCl, 25 mM NaF, 5 mM MgCl_2_, 1 mM DTT, 5 mM ATP and 0.5 mg ml^−1^ BSA in a total volume of 400 *μ*l. For inhibition experiments, the reaction mixture was preincubated with 2 mM 2-chloroadenosine, thymidine, uridine, thymine, adenine or GCV for 5 min at 37°C. HSVtk activity was determined by incubating 0.175±0.025 MBq [^18^F]FHPG in the reaction mixture at 37°C for 20 min. The reaction was stopped by loading two times 50 *μ*l of reaction mixture on two Whatman DE-81 filters. The negatively charged phosphorylated [^18^F]FHPG is bound to these filters. One filter was washed three times for at least 2 min with ammonium formate and three times with 95% ethanol to remove unreacted [^18^F]FHPG. The unwashed filter (reflects both unchanged and phosphorylated [^18^F]FHPG) was used to normalise the activity bound on the washed filter. Radioactivity of the filters was counted with a gamma counter. [^18^F]FHPG phosphorylation in the presence of a transport inhibitor was calculated by dividing the radioactivity (cpm) of the washed filters by the radioactivity of the unwashed filters: (*A*_phos_/*A*_tot_)_inhib_. Background correction was obtained from control reactions that were performed in the reaction mixture without the crude cell lysate: (*A*_phos_/*A*_tot_)_blanco_. In each experiment, [^18^F]FHPG phosphorylation in the reaction mixture that was not preincubated with an inhibitor (*A*_phos_/*A*_tot_)_cont_ was used as a reference point. Results are reported as percentages inhibition of [^18^F]FHPG phosphorylation by the inhibitors according to the formula:


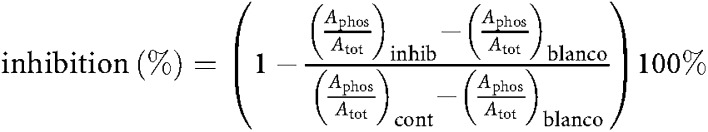


For each inhibitor, three independent experiments were performed in duplicate. Statistical analysis was performed on the raw data (*n*=6), using the two-sided unpaired student's *t*-test. *P*<0.05 was considered significant.

### Animal model

To confirm the results of our *in vitro* experiments in an *in vivo* model, the effect of blocking the purine nucleobase carrier with adenine on the [^18^F]FHPG uptake was investigated in tumour-bearing rats. To this purpose, tumours were grown in female nude rats (HSD Han RNU rnu; Harlan, the Netherlands; body weight, 140–200 g) by injection of C6 or C6tk cells. Before injection, C6 and C6tk cells (2.5 × 10^6^ cells/0.1 ml of DMEM/5% FCS) were mixed with 0.1 ml of Matrigel. Subsequently, the C6 and C6tk cell suspensions were injected into the left and right flank, respectively, close to the forelegs. At 9 or 10 days after injection of the tumour cells, a solid tumour nodule of approximately 1.5 cm in diameter had grown in each flank. In this experimental setting, the animal carried both an HSVtk containing tumour and a control tumour, which minimises the effect of interindividual variation, because each animal serves as its own control. All studies were carried out in compliance with the national law and the local ethical guidelines for animal experiments. The protocols were approved by the Animal Ethics Committee of the Groningen University.

### Accumulation of [^18^F]FHPG in tumours

The tumour-bearing nude rats were anaesthetised by an intraperitoneal injection of sodium pentobarbital (60 mg kg^−1^ body weight). During the experiment, the rats were kept warm by heating pads. Rats in the adenine group (*n*=3) were pretreated with a 1 ml bolus injection of a 33.8±5.4 mM solution of adenine in 0.9% NaCl in the tail vein, 20 min prior to tracer injection. Immediately after the bolus injection, the rats were given a constant infusion of the adenine solution (0.08 ml min^−1^) via the tail vein canula until the end of the experiment. Rats in the control group (*n*=3) were treated similarly with 0.9% NaCl only. For the determination of the adenine serum concentration, in one rat, blood samples were drawn from a carotis canula at different time intervals (5, 15, 25, 40, 55 and 70 min after the bolus adenine injection). HPLC analysis, using the procedure described by Eells and Spector (1983), revealed that a steady-state adenine serum concentration of approximately 2 mM was achieved. [^18^F]FHPG (37±18 MBq in 0.2–0.6 ml of HPLC eluent) was injected via the tail vein canula of the rats by interrupting the adenine infusion for a few seconds. After 1 h of tracer distribution, the rats were killed by extirpation of the heart. Tumours were dissected, weighed and the accumulated radioactivity was measured with a gamma counter. Tracer accumulation is expressed as the ratio between the [^18^F]FHPG accumulation per gram tissue in the C6tk tumour and the C6 tumour. Statistical analysis was performed using the two-sided unpaired student's *t*-test. *P*<0.05 was considered significant.

## RESULTS

### Accumulation of [^18^F]FHPG in tumour cells

**Figure 2:**
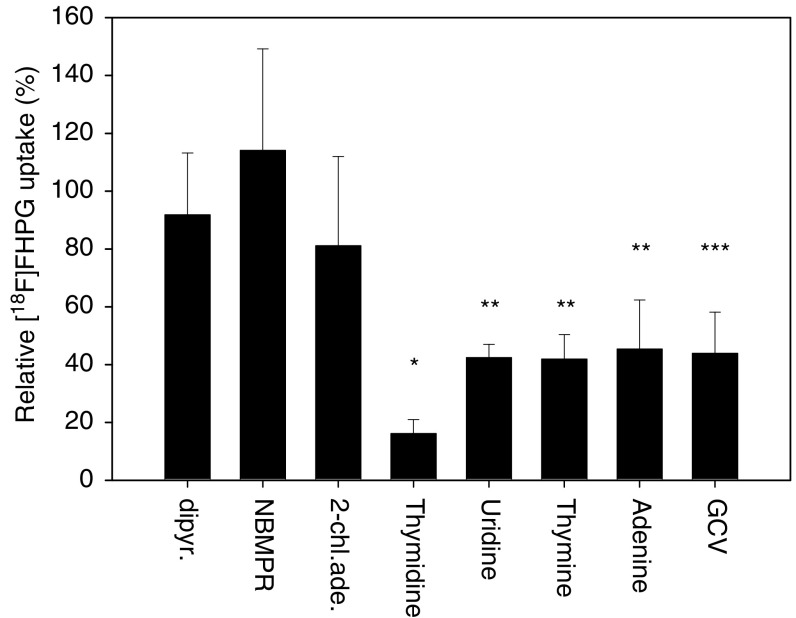
The relative [^18^F]FHPG uptake in HSVtk expressing C6tk cells. Cells were preincubated with 5 *μ*M dipyridamole, 5 *μ*M NBMPR, 2 mM 2-chloroadenosine, 2 mM thymidine, 2 mM uridine, 2 mM thymine, 2 mM adenine, or with 2 mM GCV for 20 min at 37°C. After the cells were incubated with 2.4±1.1 MBq of [^18^F]FHPG for 20 min, tracer uptake was determined. The uptake of [^18^F]FHPG in untreated C6tk cells was used as a control. Results are reported as the percentage [^18^F]FHPG uptake relative to the [^18^F]FHPG uptake in C6tk cells that were not pretreated with a transport inhibitor. Error bars represent the standard deviation. Statistical analysis was determined by the two-sided unpaired student's *t*-test. Significant differences between cells pretreated with an inhibitor and control cells are indicated with symbols: ^*^P<0.001; ^**^P<0.005; ^***^P<0.05.

To elucidate the transport mechanism, uptake of [^18^F]FHPG was studied in the presence of known inhibitors of various nucleoside and nucleobase transport systems. In the absence of inhibitors, the accumulation of [^18^F]FHPG was approximately 14 times higher in C6tk cells than in HSVtk negative C6 cells (0.140±0.043%ID/10^6^ cells for C6Tk cells and 0.011±0.004%ID/10^6^ cells for C6 cells) after 20 min of incubation with [^18^F]FHPG. Dipyridamole and NBMPR, inhibitors of the equilibrative facilitated diffusion nucleoside transport systems, did not significantly affect the [^18^F]FHPG uptake. The substrates of the concentrative sodium-dependent nucleoside transporters thymidine and uridine significantly decreased tracer uptake by 84±5 and 58±5%, respectively, whereas 2-chloroadenosine, which is also a substrate of this nucleoside transporter, had no significant effect on the [^18^F]FHPG uptake. After incubation with the nucleobase transport inhibitors thymine and adenine, a significant decline in tracer uptake of 58±8 and 55±17% was observed, respectively. Incubation with GCV resulted in a decline in tracer uptake of 59±14% (Figure 2

).

## ENZYME ASSAY

To discriminate whether the reduction in [^18^F]FHPG uptake was due to inhibition of FHPG membrane transport or inhibition of the HSVtk enzyme, an enzyme assay was performed. Thymidine, uridine and GCV are substrates of HSVtk and competition with FHPG could explain the decline in tracer uptake as well.

**Figure 3:**
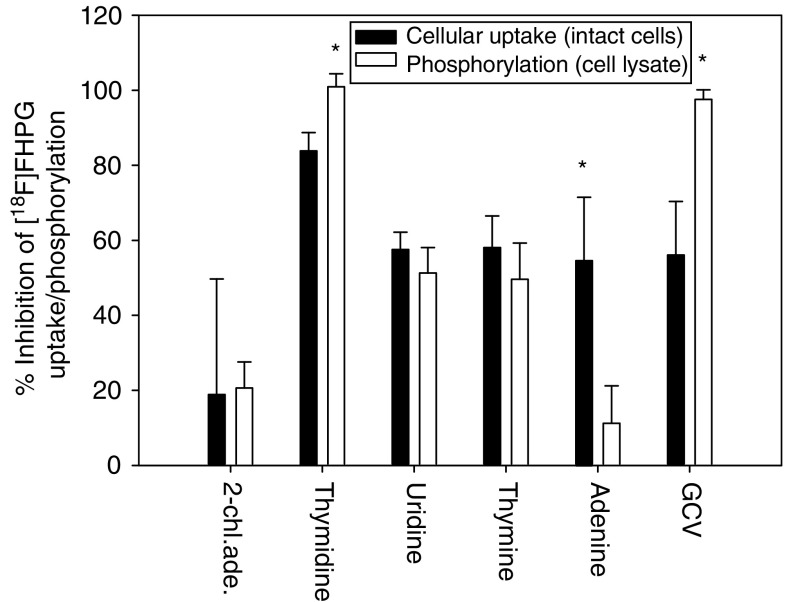
The percentage of the inhibition of the phosphorylation and cellular uptake of [^18^F]FHPG after incubation with the inhibitors in both intact cells and crude cell lysate, respectively. For the conditions of the inhibition experiments with the intact cells, see legend to Figure 2. For inhibition experiments with crude cell lysate, a standard reaction mixture was preincubated with 2 mM 2-chloroadenosine, thymidine, uridine, thymine, adenine or GCV for 5 min at 37°C. HSVtk activity was determined by incubating 0.175±0.025 MBq [^18^F]FHPG in the reaction mixture at 37°C for 20 min. In each experiment, [^18^F]FHPG phosphorylation in the reaction mixture that was not preincubated with an inhibitor, was set at 100% (control value). Results are reported as percentages inhibition of [^18^F]FHPG phosphorylation by the inhibitors. Error bars represent the s.d. Statistical analysis was determined by the two-sided unpaired student's *t*-test. Significant differences between inhibition of tracer uptake and inhibition of phosphorylation are indicated with the symbol ^*^(*P*<0.005).

After preincubation of the crude cell lysate with a competitor, a decline in [^18^F]FHPG phosphorylation was seen of 21±7% for 2-chloroadenosine, 101±3% for thymidine, 51±7% for uridine, 50±10% for thymine, 11±10% for adenine and 98±3% for GCV. Figure 3

shows the inhibition of cellular uptake and phosphorylation of [^18^F]FHPG by the inhibitors. A significant difference between the decline in [^18^F]FHPG phosphorylation in the enzyme assay and [^18^F]FHPG uptake in intact cells is seen after incubation with thymidine, adenine or GCV. Adenine blocks a great part of the [^18^F]FHPG uptake in intact cells, but hardly affects phosphorylation of [^18^F]FHPG in the crude cell lysate. Thymidine and GCV, on the other hand, almost completely inhibit [^18^F]FHPG phosphorylation in the enzyme assay, but inhibit tracer uptake in intact cells to a smaller extent.

### Accumulation of [^18^F]FHPG in tumours

To confirm the results of the *in vitro* experiments, the effect of inhibition of the purine nucleobase carrier with adenine on the [^18^F]FHPG uptake was studied in tumour-bearing rats. At 1 h after tracer injection, tumours were excised and the [^18^F]FHPG content in the tumours was determined by *ex vivo* gamma counting.

In control rats, 3.0±0.5 times more [^18^F]FHPG had accumulated in the HSVtk expressing C6tk tumour than in the C6 control tumour. After increasing the adenine plasma levels to approximately 2 mM via adenine infusion, the difference in tracer uptake between the positive and negative tumour was completely abolished (ratio: 1.0±0.2). The decrease in tumour uptake ratio as a result of adenine treatment was statistically significant (*P*<0.005).

## DISCUSSION

The aim of this study was to explore whether [^18^F]FHPG PET could be a suitable method to assess the pharmacokinetics of GCV. It has already been shown that both FHPG and GCV are phosphorylated by HSVtk to a similar extent (Martin *et al*, 1986). However, the pharmacokinetics are not only determined by phosphorylation but also by membrane transport. Whether FHPG also mimics GCV in its transport across the cell membrane remained to be investigated. In this study, we aimed to investigate whether FHPG and GCV are carried across the cell membrane by the same transporter. To this purpose, the accumulation of (^18^F]FHPG in an HSVtk expressing cell line and in HSVtk expressing tumours was examined. Inhibition experiments were performed with different inhibitors of nucleoside and nucleobase transport systems. In the literature, two classes of nucleoside transporters have been described: the equilibrative, nonconcentrative, facilitated diffusion systems and the concentrative, Na^+^- and energy-dependent systems. In the case of the equilibrative nucleoside transporters (ENT), the flux of nucleoside molecules across the membrane is driven solely by the concentration gradient. Two subtypes have been described. The equilibrative sensitive transporter (ENT1) is sensitive to low nanomolar concentrations of NBMPR. The equilibrative insensitive type (ENT2) is relatively insensitive to NBMPR. Both subtypes are inhibited by nanomolar concentrations of dipyridamole (Baldwin *et al*, 1999; Cass *et al*, 1999; Hyde *et al*, 2001). According to the literature, C6 rat glioma cells express nucleoside transporters primarily of the ENT 2 subtype (Sinclair *et al*, 2000). In our C6 glioma cell line, we found no contribution of equilibrative, facilitated nucleoside transport systems to the transport of FHPG. Dipyridamole and NBMPR, both inhibitors of this transport system, did not significantly affect [^18^F]FHPG uptake.

The concentrative nucleoside transporters (CNT) can be subdivided into three main types on the basis of their substrate selectivities. The first type, *cit*, transports preferentially pyrimidine nucleosides. The second type, *cif*, accepts purine nucleosides and uridine. The third type, *cib*, accepts both purine and pyrimidine nucleosides. All three types are typically insensitive to NBMPR (Baldwin *et al*, 1999).

In our cell line, FHPG permeation was not significantly inhibited by the purine nucleoside 2-chloroadenosine, which is also a substrate of concentrative sodium-dependent nucleoside transport. This indicates that transport of FHPG does not depend on the *cif* or *cib* carrier. The pyrimidine nucleosides thymidine and uridine, on the other hand, both decreased [^18^F]FHPG uptake. This could be evidence for transport of FHPG via the *cit* carrier. Thymidine and uridine, however, are not only substrates of the *cit* nucleoside transporter but also of the HSVtk enzyme. Competition of these nucleosides with FHPG for the HSVtk enzyme might have caused the observed effects. To discriminate between the reduction in [^18^F]FHPG uptake via inhibition of FHPG membrane transport and inhibition of the HSVtk enzyme, an enzyme assay was performed. In the enzyme assay, the decline in [^18^F]FHPG phosphorylation after preincubation with 2-chloroadenosine and uridine corresponded well with the decline in tracer uptake as seen in intact cells. This indicates that the decline in [^18^F]FHPG uptake as seen in the intact cells is caused by competition of these compounds with [^18^F]FHPG as substrates for the HSVtk enzyme and not by inhibition of membrane transport. In the case of thymidine, [^18^F]FHPG phosphorylation is completely inhibited in the enzyme assay, whereas the [^18^F]FHPG uptake in the intact cells is inhibited to a smaller extent. This probably means that membrane transport of thymidine is the rate-limiting step and as a consequence only low intracellular levels of thymidine were achieved in our experiments. Therefore, the decline in [^18^F]FHPG uptake in intact cells is completely due to competition of thymidine with [^18^F]FHPG as a substrate for the HSVtk enzyme. Additionally, the fact that the decline in tracer uptake in intact cells is smaller than the reduction in FHPG phosphorylation in the presence of thymidine, indicates that FHPG is transported by another carrier than thymidine. Thus, in our cell line, we found no evidence for concentrative nucleoside transport for FHPG.

Nucleobase transport has been extensively studied. In certain mammalian cells, separate carriers for hypoxanthine (guanine) and adenine have been assumed to exist. In contrast, results of other cell studies have suggested the existence of a single carrier for purine nucleobase transport. In human erythrocytes purine nucleobases share a common facilitated transport system, which is functionally distinct from the nucleoside transporter (Domin *et al*, 1988).

To investigate the contribution of nucleobase systems to the transport of FHPG, competition experiments with a pyrimidine nucleobase (thymine) and a purine nucleobase (adenine) were carried out. According to literature, the presence of at least two nucleobase transporters is suggested in C6 rat glioma cells: NBT 1 and NBT 2 (Sinclair *et al*, 2000). Thymine and adenine caused a significant decline in tracer uptake of 58 and 55%, respectively. This suggests that FHPG is transported by purine and pyrimidine nucleobase carriers.

However, the enzyme assays with crude cell lysate showed that thymine inhibited [^18^F]FHPG phosphorylation to a similar extent as it inhibited cellular uptake of the tracer in intact cells. One of the explanations for this observation could be a disturbed balance between thymine metabolism and thymidine catabolism due to the enormous excess of exogenous thymine. Normally, the following reaction, catalysed by thymidine phosphorylase, maintains the balance of the intracellular nucleotide pool:


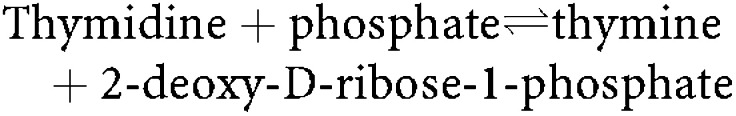


Although the reaction is reversible, nucleic acid homeostasis depends mainly on the catabolic reaction (Brown and Bicknell, 1998; Murray *et al*, 2002). The enormous increase in thymine levels might reverse this balance. An increase in thymidine levels results in an increased inhibition of [^18^F]FHPG phosphorylation in the crude cell lysate via competition for the HSVtk enzyme, and thus a decreased uptake of the tracer in intact cells.

The fact that no clear difference was seen between the decline in [^18^F]FHPG phosphorylation in the crude cell lysate and the decline in [^18^F]FHPG uptake in intact cells indicates that there is no inhibition of [^18^F]FHPG membrane transport by thymine. Therefore, no evidence for pyrimidine nucleobase transport for FHPG is found in our cell line.

In the case of adenine, there was no inhibition on the [^18^F]FHPG phosphorylation in the enzyme assay. Together with the fact that a clear difference was seen between the decline in [^18^F]FHPG uptake in crude cell lysate and in intact cells, this result indicates that the reduction in [^18^F]FHPG uptake as seen in intact cells was indeed due to inhibition of the [^18^F]FHPG transport. Also *in vivo*, a complete block of the [^18^F]FHPG uptake in HSVtk positive tumours could be accomplished by infusion with the purine nucleobase adenine. It follows from the above that FHPG enters the C6 cells exclusively via the purine *nucleobase* carrier.

The goal of our experiments was to investigate if FHPG mimics GCV in its transport across the cell membrane. Membrane transport of GCV has been studied in different cell lines. In human erythrocytes, GCV permeates the membrane both by the purine nucleobase carrier and by the nucleoside transporter (Mahony *et al*, 1991). In human mammary carcinoma (MCF7) cells, nucleobase systems showed no significant contribution to GCV uptake, as only transport via the nucleoside transport systems was seen (Haberkorn *et al*, 1998). In rat Morris hepatoma (MH3924A) cells, GCV was also transported by the nucleoside transport systems (Haberkorn *et al*, 1997). So, in general, GCV is primarily transported by the *nucleoside* transport systems. In our experiments, we found only partial inhibition of [^18^F]FHPG uptake in intact cells by GCV, whereas GCV completely blocked [^18^F]FHPG phosphorylation in the enzyme assay. These results are in agreement with the results of Haberkorn *et al* (1998), who found that GCV enters the cell relatively *slowly*, mainly via the nucleoside transport system. As a result of this slow GCV transport, probably only low intracellular levels of GCV were achieved in our experiments. Therefore, in intact cells, GCV only partly inhibited [^18^F]FHPG phosphorylation. FHPG membrane transport was probably unaffected by GCV, because it utilises a different transport system.

Thus, in our C6 tumour model, FHPG has proven to be transported by the purine nucleobase carrier. The type transporters that are expressed in a cell can vary between tumour cells and therefore in other tumour cell lines, other transporters may play a role in the uptake of FHPG. However, our results show that membrane transport of FHPG is different of that of GCV at least in one cell line and we expect this to be the case also in other tumour cell lines. Since GCV and FHPG in principle can be transported into the cell by different carriers, FHPG is not a good indicator for GCV membrane transport and therefore not suitable to monitor GCV pharmacokinetics, irrespective of which transporters are expressed in the tumour cell.

The fact that FHPG – in contrast to GCV – is primarily transported by the purine *nucleobase* carrier might be explained by the difference in the chemical structures of FHPG and GCV. It has been shown for pyrimidine nucleoside probes that modification at the 3′-position, loss of a portion of the sugar ring, and lack of conformational flexibility can decrease the affinity for the nucleoside transporters and can result in a decreased transport into the cells (Gati *et al*, 1984). GCV has a relatively strong similarity to nucleosides due to the presence of two hydroxymethyl groups on its side chain and, therefore, can be transported by a nucleoside transporter (Haberkorn *et al*, 1998). In the structure of FHPG, the second hydroxyl group is missing (Figure 1) and as a result the affinity for the nucleoside transporter could be lost.

The second hydroxyl group is also missing in the structure of acyclovir (ACV), another acycloguanosine analogue, which is phosphorylated by HSVtk. The HSVtk phosphorylation rate of FHPG is intermediate between that of GCV and ACV (Martin *et al*, 1986). In human erythrocytes, ACV enters the cells mainly via the purine nucleobase carrier (Mahony *et al*, 1988). Our results indicate that in our C6 cell line model FHPG is also mainly transported by the purine nucleobase carrier. Owing to this resemblance in transport across the cell membrane and phosphorylation characteristics, FHPG might be suitable to determine ACV pharmacokinetics. This could be of clinical importance because the GCV dose required for tumour regression is immunosuppressive, whereas the required ACV dose is relatively nontoxic. Thus far, ACV has not been used in a clinical HSVtk/ACV gene therapy setting, because of the lower affinity for the HSVtk enzyme as compared to GCV (Black *et al*, 2001). However, Black *et al* (2001) recently developed HSVtk mutants with increased sensitivity towards ACV. These mutants could bring HSVtk-mediated activation of ACV to a clinically relevant level and, as a consequence, HSVtk/ACV gene therapy could become of clinical importance. Noninvasive imaging of ACV pharmacokinetics could also play a role in other herpes related diseases, like for example, Herpes Simplex Encephalitis (HSE), which is treated with high doses of ACV. Case reports indicate that HSE may relapse after ACV therapy. This relapse could, among others, be related to persistence of infection (Skoldenberg, 1996). In addition, in immunodeficient patients, ACV-resistant HSV isolates have been identified (Roos, 1999). Additional studies are warranted to show feasibility of [^18^F]FHPG PET to assess the pharmacokinetics of ACV at the onset of ACV treatment, which may allow prediction of treatment outcome.

## CONCLUSION

Our results show that the transport of FHPG in C6 cells exclusively occurs by the purine nucleobase carrier. Thus, the transport mechanism of FHPG appears to resemble that of ACV, rather than GCV. As a consequence, FHPG cannot be used to measure GCV pharmacokinetics. Whether [^18^F]FHPG is suited to assess the pharmacokinetics of the prodrug ACV or to predict the efficacy of ACV treatment in HSVtk/ACV gene therapy or other herpes-related diseases remains to be investigated.
